# Occurrence of Relative Bradycardia and Relative Tachycardia in Individuals Diagnosed With COVID-19

**DOI:** 10.3389/fphys.2022.898251

**Published:** 2022-05-10

**Authors:** Aravind Natarajan, Hao-Wei Su, Conor Heneghan

**Affiliations:** Fitbit Research, Google Inc., San Francisco, CA, United States

**Keywords:** digital health, heart rate variability, COVID-19, heart rate, bradycardia, tachycardia

## Abstract

The COVID-19 disease caused by the Severe Acute Respiratory Syndrome Coronavirus 2 (SARS-CoV-2) has become one of the worst global pandemics of the century. Wearable devices are well suited for continuously measuring heart rate. Here we show that the Resting Heart Rate is modified for several weeks following a COVID-19 infection. The Resting Heart Rate shows 3 phases: 1) elevated during symptom onset, with average peak increases relative to the baseline of 1.8% (3.4%) for females (males), 2) decrease thereafter, reaching a minimum on average ≈13 days after symptom onset, and 3) subsequent increase, reaching a second peak on average ≈28 days from symptom onset, before falling back to the baseline ≈112 days from symptom onset. All estimates vary with disease severity^1^.

## 1 Introduction

Cardiac complications are known to be associated with the COVID-19 disease caused by the novel Severe Acute Respiratory Syndrome—Coronavirus 2 (SARS-CoV-2) (Cizgici et al., 2020; Huang et al., 2020; Kochi et al., 2020; Long et al., 2020; Shi et al., 2020; Siripanthong et al., 2020; Wang et al., 2020; Coromilas et al., 2021). An unusual feature that is sometimes seen in patients diagnosed with COVID-19 is the appearance of bradycardia, i.e., slow heart rate, or heart rate not increasing as expected with body temperature (Ikeuchi et al., 2020; Capoferri et al., 2021; Douedi et al., 2021; Oliva et al., 2021). Amaratunga et al. (2020) found bradycardia in a study of 4 patients with confirmed COVID-19, with minimum pulse rates in the range 42–49 beats per minute. Amir et al. (2021) reported 6 cases of bradycardia among patients diagnosed with COVID-19, with 4 patients developing complete atrioventricular block. Elikowski et al. (2021) presented clinical data of 19 patients diagnosed with COVID-19 who exhibited sinus bradycardia, in some cases showing heart rates as low as 32 bpm during daily hours. Srinivasan et al. (2021) discussed 6 cases of patients who were diagnosed with COVID-19 and admitted with normal sinus rhythm, and who subsequently developed sinus bradycardia with daytime heart rates ranging from 35–48 bpm. In a study of 97 patients with a non-severe presentation of COVID-19, Zhou et al. (2021) found significant sinus bradycardia (below 50 bpm) in 7.2% of cases.

In addition to bradycardia, COVID-19 is known to cause a number of other cardiac anomalies. In a study of 140 individuals diagnosed with COVID-19 and 281 individuals diagnosed with influenza, Lampert et al. (2021) found that diminished QRS amplitude was dynamic during the course of illness, and was an independent predictor of mortality with both COVID-19 and influenza, but was more prevalent in the case of COVID-19. Cardiac injury, heart failure, and arrhythmias have been recorded in patients diagnosed with COVID-19 (Linschoten et al., 2020; Shi et al., 2020; Coromilas et al., 2021; Sahranavard et al., 2021). Guo et al. (2020) reported ventricular tachycardia (VT) or ventricular fibrillation (VF) in 11 out of 187 patients with confirmed COVID-19 and found that elevated levels of troponin T were correlated with VT/VF. Inflammatory damage due to cytokines has been suggested as a possible explanation for cardiac involvement with COVID-19 (Bhatla et al., 2020; Dherange et al., 2020; Peigh et al., 2020; Elikowski et al., 2021). In a retrospective analysis of 3,970 patients admitted with COVID-19 and 1,420 patients admitted with influenza, Musikantow et al. (2021) found similar incidences of atrial fibrillation/atrial flutter with COVID-19 (10%) and influenza (12%). The presence of atrial fibrillation was associated with increased mortality in both COVID-19 and influenza (Musikantow et al., 2021). New-onset atrial fibrillation was found to be influenced most by markers of inflammation such as Interleukin 6 and C-reactive protein (Musikantow et al., 2021). Pardo Sanz et al. (2021) found that new-onset atrial fibrillation is associated with worse outcomes than in patients with existing atrial fibrillation.

The presence of atrial fibrillation in patients with severe COVID-19 was found to be 6 times higher compared to patients with non-severe COVID-19 (Li et al., 2021). Possible mechanisms for the development of atrial fibrillation in patients diagnosed with COVID-19 have been discussed by Stone et al. (2020) and Gawałko et al. (2020). Turagam et al. (2020) studied the occurrence of malignant arrhythmias and found that VT/VF and AV block were more commonly associated with mortality, and that tachyarrhythmias such as VT/VF occurred in the setting of severe metabolic stress. In a comparison of the mortality group with the discharged group, it was found that serum creatinine, peak troponin, C-reactive protein, Procalcitonin, and interleukin 6 levels were all significantly elevated in the mortality group (Turagam et al., 2020). COVID-19 sometimes presents with rare symptoms such as syncope (Oates et al., 2020).

Commercially available wearable devices have been shown to be useful in early detection of COVID-19 and for monitoring symptoms (Miller et al., 2020a; Miller et al., 2020b; Mishra et al., 2020; Natarajan et al., 2020b; Natarajan et al., 2021; Quer et al., 2021). Radin et al. (2021) studied resting heart rate (henceforth RHR) data from Fitbit devices to investigate long term changes following symptom onset. RHR is typically elevated around symptom onset. We use the term “relative” to indicate that the RHR is elevated/decreased relative to the baseline value for that individual, although the RHR is not necessarily above/below the clinical threshold guideline (Ostchega et al., 2011). They also found the RHR exhibits a dip which we refer to as transient relative bradycardia provided the RHR is below the baseline value. The RHR dip was followed by a second elevated RHR peak. They found that the RHR was elevated for up to 79 days from symptom onset.

In this article, we obtain results consistent with the findings of Radin et al., and expand upon existing work in a number of ways. We study a much larger sample size than previously considered. We investigate how the RHR changes, for male and female individuals, and for individuals with severe, mild or asymptomatic presentations of COVID-19. We also consider individuals diagnosed with the seasonal influenza (henceforth “flu”). We tabulate the expected amplitudes of the maxima/minima, as well as the time taken to reach these maxima/minima, and the estimated widths of the peaks/troughs. We examine how these parameters vary with age, sex, and disease severity. We also study heart rate variability and respiratory rate and how these metrics vary with time.

## 2 Methods

### 2.1 Survey Data

**FIGURE 1:**
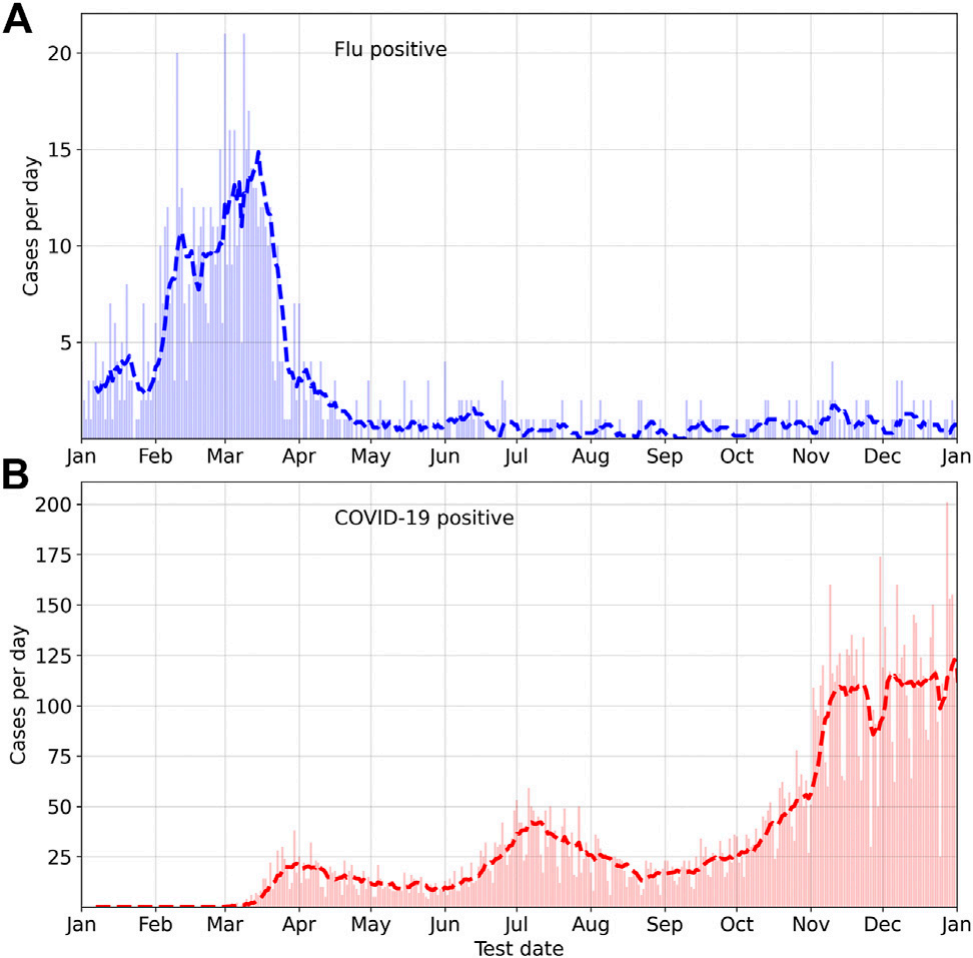
Incidence of Flu and COVID-19 in the year 2020, from the Fitbit COVID-19 survey.

The Fitbit COVID-19 survey was conducted from 21 May 2020 to 10 June 2021, and collected data from participants residing in the United States or Canada. Participants provided information on whether they were diagnosed with COVID-19 or flu, as well as the test date, symptoms, and the start date of symptoms. Participants could optionally provide information about their age, sex, body mass index, and information on underlying conditions. Individuals diagnosed with COVID-19 also indicated the severity of the disease which could be 1) severe, indicating that they required hospitalization, 2) mild, indicating that they recovered at home, or 3) asymptomatic. The survey and associated marketing and recruitment materials were approved by an Institutional Review Board (Advarra). The participants provided electronic informed consent for their data to be used for research. In this study, we consider data from Fitbit users who reported testing positive for COVID-19 in the date range March 1—31 Dec 2020, as well as users who reported testing positive for flu in the date range January 1—31 Dec 2020. There were 11,918 participants who tested positive for COVID-19 (mean age = 40.8 years, std. dev. = 12.4 years, 79.0% female) in our dataset, and 865 participants who tested positive for flu (mean age = 41.7 years, std. dev. = 13.3 years, 78.2% female). We randomly selected 1,000 users who did not report a positive test for either COVID-19 or flu as a control group (mean age = 45.3 years, std. dev. = 13.9 years, 71.6% female). Table 1 shows the prevalence of symptoms (self reported) for COVID-19 and flu, for male and female participants (for COVID-19, we also separate by disease severity). Some symptoms such as fatigue, headache, and body ache are common for both COVID-19 and flu. By contrast, a decrease in taste and/or smell is more likely in the case of COVID-19 (72.6% female, 59.8% male) compared to flu (21.2% female, 11.6% male). Fever is more common with flu (81.3% female, 76.8% male) compared to COVID-19 (51.4% female, 58% male). Figure 1 shows the distribution of positive cases for flu and COVID-19, where the horizontal axis is the test date, and only positive cases are shown. Cases of flu peaked in March 2020, while COVID-19 cases peaked much later.

**TABLE 1:** Prevalence of symptoms (expressed as a fraction), for COVID-19 (mild/severe) and flu, for male and female participants.

Symptom	Mild (fem.)	Mild (male)	Severe (fem.)	Severe (male)	Flu (fem.)	Flu (male)
Fatigue	0.847	0.761	0.885	0.816	0.843	0.689
Headache	0.793	0.640	0.789	0.618	0.661	0.415
Decrease in taste/smell	0.727	0.598	0.701	0.592	0.211	0.116
Congestion	0.715	0.571	0.503	0.342	0.541	0.512
Body ache	0.689	0.666	0.793	0.684	0.818	0.677
Cough	0.652	0.627	0.784	0.730	0.731	0.616
Chills	0.532	0.557	0.713	0.632	0.734	0.604
Fever	0.501	0.563	0.749	0.796	0.813	0.768
Sore throat	0.498	0.385	0.418	0.342	0.560	0.378
Loss of appetite	0.472	0.345	0.701	0.638	0.440	0.293
Shortness of breath	0.432	0.347	0.871	0.836	0.396	0.299
Chest pain	0.401	0.284	0.687	0.533	0.334	0.244
Diarrhea	0.399	0.319	0.524	0.454	0.192	0.189
Neck pain	0.296	0.186	0.322	0.191	0.193	0.116
Hoarse voice	0.280	0.179	0.320	0.303	0.279	0.110
Eye pain	0.232	0.161	0.232	0.197	0.106	0.067
Stomach ache	0.225	0.115	0.290	0.118	0.130	0.104
Confusion	0.178	0.135	0.368	0.349	0.114	0.140
Vomiting	0.089	0.044	0.271	0.118	0.115	0.067
Rash	0.067	0.034	0.131	0.066	0.029	0.037
Swelling in fingers/toes	0.044	0.022	0.133	0.079	0.032	0.043

### 2.2 RHR Data

We measure the RHR from participants in our study, collected using Fitbit devices. RHR is computed using heart rate data during sleep when sleep data is available, and a proprietary algorithm is utilized to predict the RHR from the time series heart rate data during sleep. If sleep data is unavailable, RHR is computed using wake time heart rate data, at times when no activity is detected. The RHR is also processed with a Kalman filter which serves to smooth the waveform. The RHR as defined by Fitbit is the value closest to the heart rate measured when lying down just before waking up in the morning. Details of how Fitbit measures resting RHR may be found in Russell et al. (2019). Note that the RHR is not the minimum value of heart rate.

### 2.3 Time Variation of the RHR Data

**FIGURE 2:**
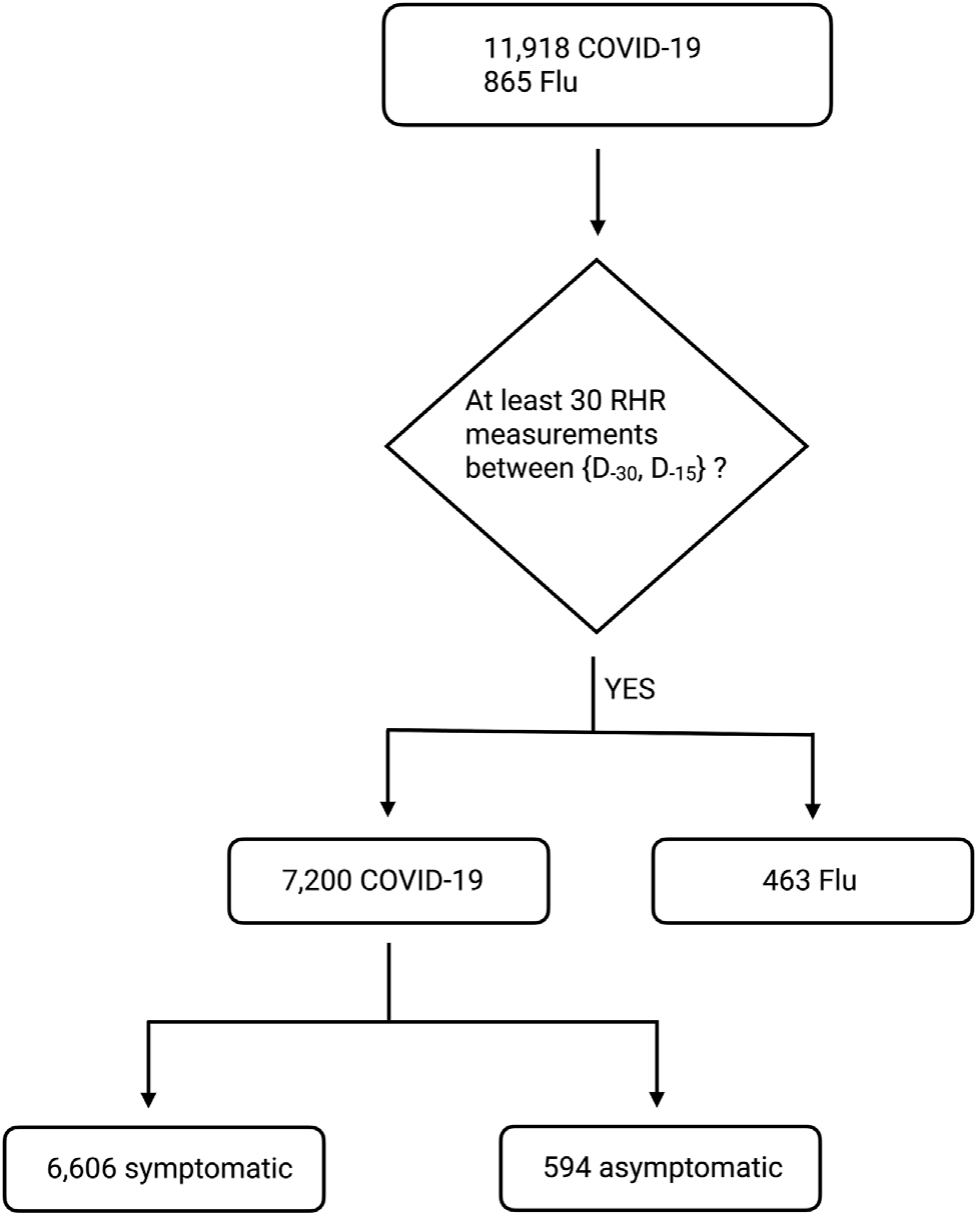
Block diagram showing the number of survey participants with COVID-19 or flu. We start with all participants with diagnosed COVID-19 between {2020-03-01, 2020-12-31}, or diagnosed flu between {2020-01-01, 2020-12-31}. Note that *D*_0_ is the date of symptom onset for symptomatic individuals (and the test date for asymptomatic individuals). We then discard individuals with fewer than 30 RHR measurements between *D*_−90_ and *D*_−15_. Our final dataset contains 463 individuals with flu, 6,606 individuals with symptomatic COVID-19, and 594 individuals with asymptomatic COVID-19.

Let *D*_0_ be the date of symptom onset for symptomatic individuals (and the test date for asymptomatic individuals). Thus for symptomatic individuals, *D*_−1_ is one day prior to the appearance of symptoms, and *D*_+1_ is one day post appearance of symptoms. We computed the mean value ⟨*RHR*⟩ for each individual by averaging the RHR values from *D*_−90_ to *D*_−15_. We discard data from participants who have fewer than 30 measurements in this time window. With this data sufficiency condition, our dataset contains 463 individuals with the flu, and 7,200 individuals with COVID-19 (6,606 symptomatic, 594 asymptomatic, see Figure 2). We compute the fractional change in RHR *ξ* from days *D*_−14_ to *D*_+180_ as:

$$\xi \left(d\right)=\frac{RHR\left(d\right)-\langle RHR\rangle }{\langle RHR\rangle },$$ (1)

where *d* is a variable indicating day index.

### 2.4 Controlling for the Seasonality of Resting Heart Rate

The RHR has a known seasonal modulation. In a study of 200,000 individuals wearing Fitbit devices and residing in the United States, Quer et al. (2020) found a change in the population’s average RHR by 2 beats per minute (bpm). In the Northern hemisphere, the RHR peaks in the first week of January and reaches its minimum at the end of July.

Since all participants in our study reside in the United States or Canada, we assume that they are subject to the same seasonal trends. We use the control group to estimate how RHR varies with the time of year, by applying Eq. 1 to obtain *ξ*_control_ as follows:

1) Randomly sample a date from the COVID-19 or flu distribution and set to *D*_0_ (date of symptom onset).
2) Compute *ξ*_control_(*d*) using Eq. 1 from the control group for dates *d* relative to *D*_0_.

We may then subtract out the seasonality to find the effect of illness on RHR:

$$\Delta \xi =\xi -\xi_{\text{control},\alpha },$$ (2)

where *α* may be flu or COVID-19. Note that *ξ*_control_ must be computed twice: once for the COVID-19 distribution, and once for the flu distribution. To compute Δ*ξ*(*d*) for a given individual, we would use the appropriate *ξ*_control,*α*_ depending on whether the participant was diagnosed with flu or COVID-19.

### 2.5 Heart Rate Variability and Respiratory Rate

Heart Rate Variability, as the name implies, refers to the variance in the heart rate. Heart Rate Variability is usually a good thing (with the exception of heart arrhythmias) since a healthy heart does not beat like a metronome. Heart Rate Variability allows the cardiovascular system to adjust to physical and psychological challenges to homeostasis (Shaffer and Ginsberg, 2017). Here, we consider the root mean square of the successive differences (RMSSD) between heart beats as the HRV metric of importance. The RMSSD is computed in 5 min windows between the hours of midnight and 7 a.m. The median of the measurements is then reported. Fitbit does not measure interbeat intervals when movement is detected, and therefore Fitbit devices primarily measure HRV during sleep. Further details of how Fitbit devices compute RMSSD have been described in Natarajan et al. (2020a). For the respiratory rate calculation, we first compute the power spectral density of interbeat intervals in 5 min windows between the hours of midnight and 7 a.m. The measurements are then averaged, and the respiratory rate is estimated from the averaged power spectral density. We subtract out the seasonality of the RMSSD and respiratory rate in a similar manner to the RHR, with the addition of a median filter (with a period of 7 days). The median filter is necessary because the RMSSD and respiratory rate waveforms are not smoothed by a Kalman filter, and are hence noisier than the RHR. The effect of illness on the RMSSD and respiratory rate may be estimated as:

$$\Delta \rho =\rho -\left\langle \rho_{\text{control}}\right\rangle ,$$ (3)

where *ρ* and *ρ*_control_ may be RMSSD or respiratory rate, and the angle brackets denote the median filter. Further details of how Fitbit devices compute the respiratory rate during sleep have been described in Natarajan et al. (2021).

### 2.6 Statistical Analysis

All analyses were performed using standard Python libraries. For parameter estimation, the waveforms for *ξ* and *ξ*_control_ were linearly interpolated, and then smoothed with a Savitzky − Golay filter with a window length of 7 days, and cubic polynomial smoothing. For the mean parameters tabulated in Tables 4, 5, we used a step size of 0.2 days for the linear interpolation. The standard error of the parameter values was estimated using the jackknife technique (Miller, 1974; Efron, 1981). For the mean and jackknife error estimated parameters shown in Table 3, we used a finer step size of 0.01 days. We computed Δ*ξ* from the smoothed waveforms, and estimated the peaks/troughs by means of a peak detection algorithm. For estimation of *p* − values, we used a one sided *t* − test. For estimation of correlation, we used the Pearson *r* coefficient.

## 3 Results

### 3.1 Change in RHR Following a Flu/COVID-19 Diagnosis

**FIGURE 3:**
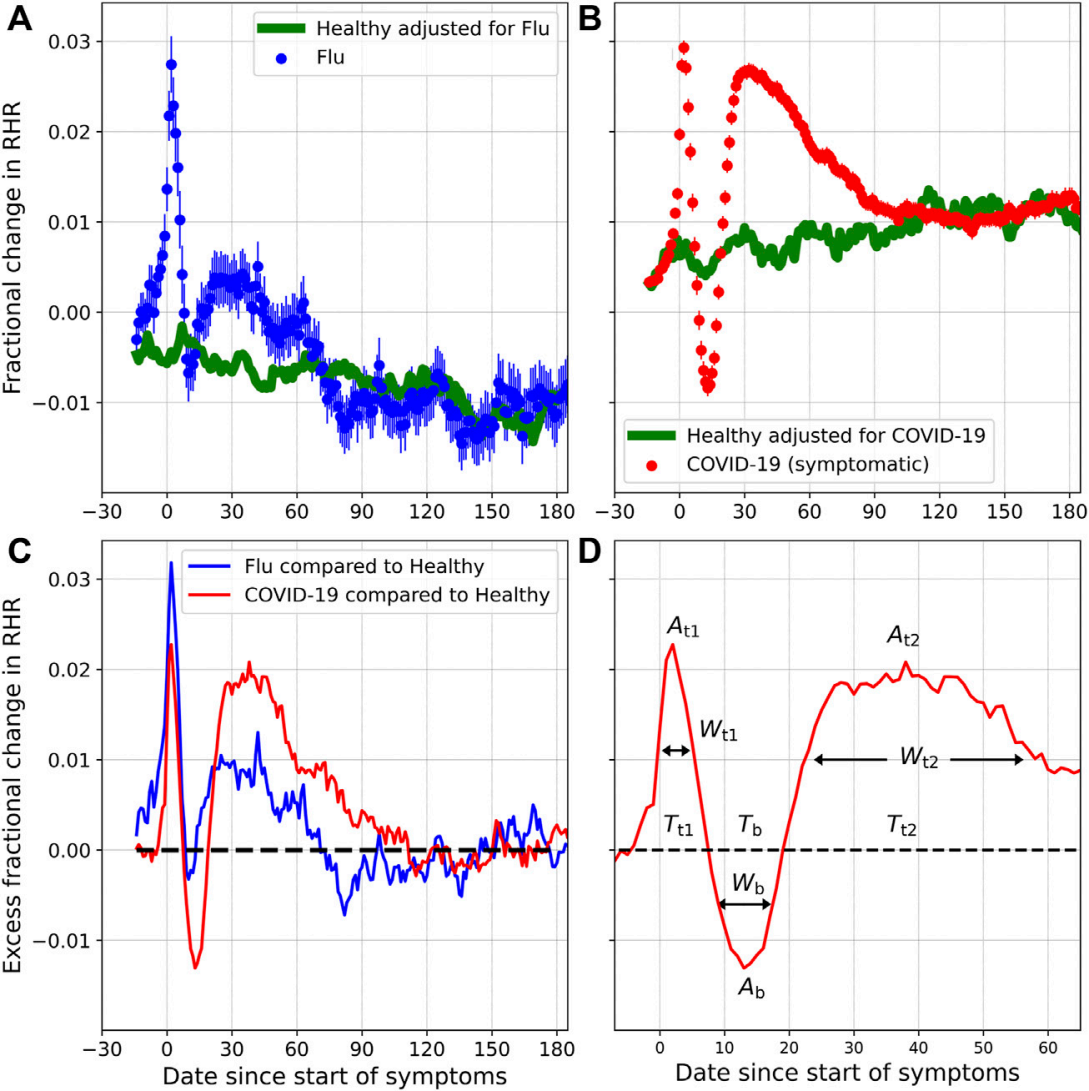
Fractional change in RHR (*ξ*), for individuals diagnosed with flu **(A)** and COVID-19 **(B)**, along with the expected seasonal variation (*ξ*_control_). **(C)** shows the excess fractional change in RHR (Δ*ξ*) for flu and COVID-19, once the contribution of seasonality has been subtracted. **(D)** shows the various parameters to be estimated.

Figure 3 shows the fractional change (*ξ*) in RHR for flu and COVID-19, for symptomatic individuals, along with the fractional change in the Control group (*ξ*_control_). Subplot 1) compares *ξ* and *ξ*_control_ for flu, while subplot 2) compares *ξ* and *ξ*_control_ for COVID-19. The horizontal axis *D*_*n*_ is the date relative to the date when symptoms first appear (*n* < 0 are prior to symptom onset, while *n* > 0 are after symptom onset. *D*_0_ is the date when symptoms first present). *ξ* and *ξ*_control_ are averaged over all individuals for a specific *D*_*n*_. Subplot 3) shows Δ*ξ*, i.e. the excess fractional change in RHR after the seasonal variation has been subtracted, while subplot 4) shows the parameters *A*_t1_, *A*_b_, *A*_t2_, *T*_t1_, *T*_b_, *T*_t2_, *W*_t1_, *W*_b_, and *W*_t2_. The parameters to be estimated are summarized in Table 2. From subplot (c), we infer the following:

1) The excess fractional change in RHR, i.e., Δ*ξ* is elevated on average during the onset of symptoms. Δ*ξ* reaches a peak value *A*_t1_ at a time *T*_t1_ days from the onset of symptoms. *W*_t1_ is the full width at half maximum. This is the first transient relative tachycardia.
2) Following the peak, Δ*ξ* decreases, reaching a minimum value *A*_b_ at a time *T*_b_ days from the onset of symptoms. *W*_b_ is the full width at half minimum (only defined when *A*_*b*_ < 0). If *A*_b_ < 0, we refer to the trough as transient relative bradycardia.
3) Following the minimum, Δ*ξ* increases again, reaching a second peak *A*_t2_ at a time *T*_t2_ days from symptom onset. *W*_t2_ is the full width at half maximum.
4) Past *T*_t2_, Δ*ξ* decreases and eventually falls to zero, indicating that the RHR variation is no longer due to illness.

**TABLE 2:** Description of parameters.

Symbol	Description
*N*	Sample size
*A*_t1_	Amplitude of the first relative tachycardia peak (no units)
*A*_*b*_	Amplitude of the relative bradycardia trough (no units)
*A*_t2_	Amplitude of the second relative tachycardia peak (no units)
*T*_t1_	Time from symptom onset to first relative tachycardia peak (days)
*T*_*b*_	Time from symptom onset to relative bradycardia trough (days)
*T*_t2_	Time from symptom onset to second relative tachycardia peak (days)
*W*_t1_	Full width at half maximum of first relative tachycardia peak (days)
*W*_b_	Full width at half minimum of relative bradycardia trough only if *A*_*b*_ < 0 (days)
*W*_t2_	Full width at half maximum of second relative tachycardia peak (days)

**FIGURE 4:**
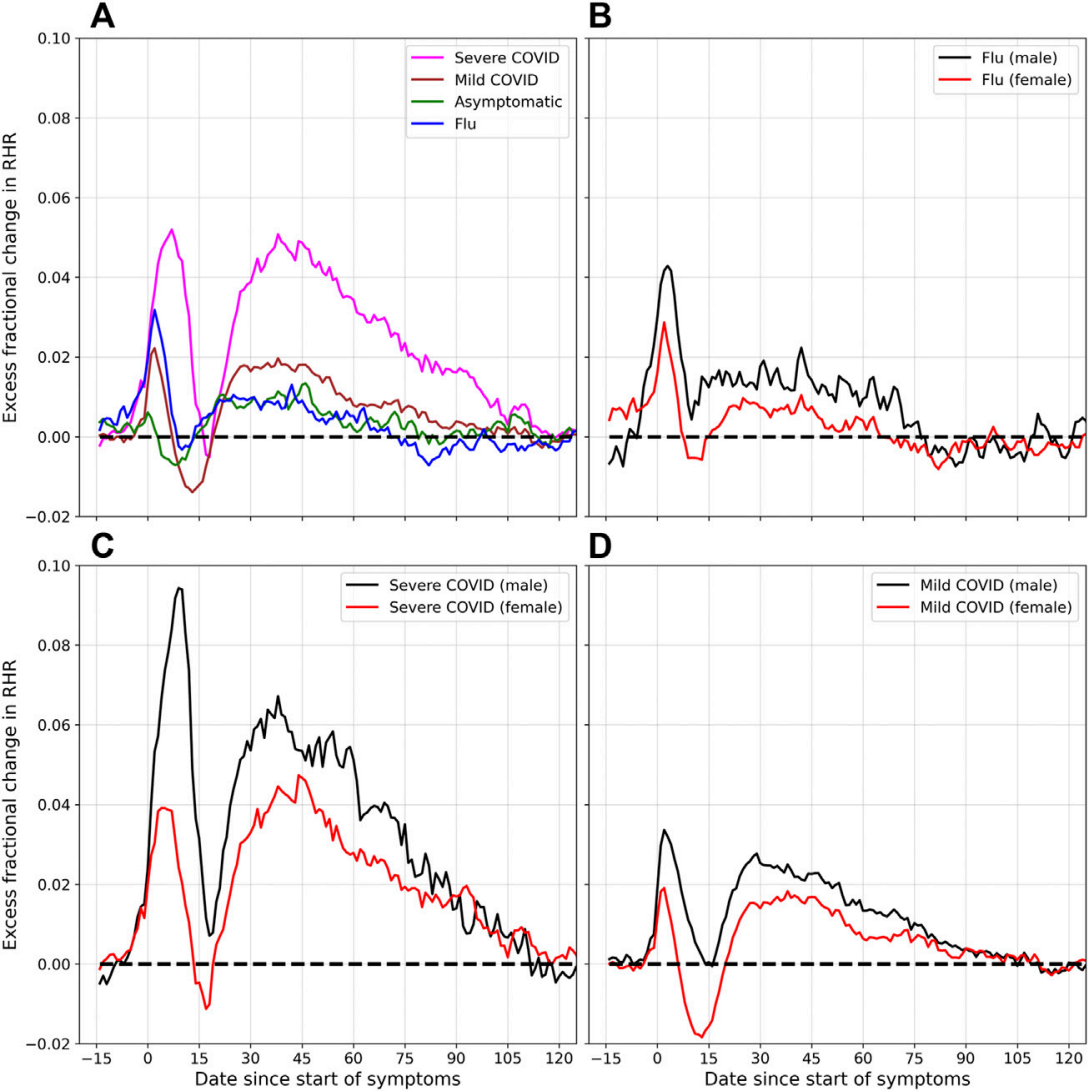
Excess fractional change in RHR (Δ*ξ*), and variation with severity and sex: **(A)** shows Δ*ξ* for severe, mild, and asymptomatic COVID-19, as well as flu. **(B)** shows Δ*ξ* for male and female individuals diagnosed with flu. **(C)** and **(D)** show Δ*ξ* for male and female participants, for the cases for severe and mild COVID-19 respectively.

Figure 4 shows the excess fractional change in RHR (Δ*ξ*) for COVID-19 and flu, for varying severity, and for male and female individuals. Subplot (a) shows the effect of severity on Δ*ξ*. The magenta curve is plotted for severe cases (i.e., cases that required hospitalization). The brown curve shows mild cases, while the green curve is plotted for asymptomatic cases. The blue curve shows Δ*ξ* for flu. The amplitudes of the two relative tachycardia peals *A*_t1_ and *A*_t2_ are much larger in the case of severe COVID-19. The bradycardia dip (*A*_b_) is more pronounced in the case of mild COVID-19. The first relative tachycardia peak amplitude (*A*_t1_) is larger for flu compared to mild or asymptomatic COVID-19, while the second tachycardia peak is similar in the cases of flu and asymptomatic COVID-19. For the cases of severe, mild, asymptomatic, and flu respectively, we find Δ*ξ* falls to zero approximately 118, 112, 79, and 71 days after the onset of symptoms (or test date for asymptomatic cases).

Subplot (b) shows the difference between male and female individuals who were diagnosed with flu. Similar to the case of COVID-19, the peak amplitudes *A*_t1_ and *A*_t2_ are higher for males compared to females. The trough amplitude *A*_*b*_ is lower for females. Subplots (c) and (d) show the variation of Δ*ξ* for male and female individuals, for severe and mild COVID-19 cases respectively. In both cases, the peak amplitudes *A*_t1_ and *A*_t2_ are higher for males, for both severe and mild cases of COVID-19. The minimum amplitude *A*_b_ on the other hand is lower in females than in males, and is more negative for mild cases of COVID-19 compared to severe cases.

Table 3 shows the estimated values of the amplitudes for the RHR minimum (*A*_b_) and the two maxima (*A*_t1_ and *A*_t2_), along with jackknife estimated standard errors. Table 4 shows the estimated values of the various parameters, for female and male participants, and for different ages. Each peak/trough is characterized by 3 parameters: the amplitude, time from symptom onset, and full width at half maximum/minimum. The 2 relative tachycardia peaks are parameterized by (*A*_t1_, *T*_*t*1_, *W*_t1_) and (*A*_t2_, *T*_*t*2_, *W*_t2_), while the minimum is parameterized by (*A*_b_, *T*_b_, *W*_b_). Note that *W*_b_ is only defined when *A*_b_ < 0 indicating relative bradycardia. Table 5 shows the estimated values of the parameters for male and female participants, for severe, mild, and asymptomatic COVID-19 presentations, as well as for flu.

**TABLE 3:** Estimation of peak amplitudes.

Disease	Sex	*N*	*A*_t1_	*A*_b_	*A*_t2_
COVID-19	F	5,183	1.78 ± 0.08	−1.75 ± 0.10	1.81 ± 0.08
COVID-19	M	1,411	3.39 ± 0.18	0.08 ± 0.18	2.80 ± 0.17
Flu	F	360	2.60 ± 0.32	−0.57 ± 0.33	0.96 ± 0.30
Flu	M	103	4.28 ± 0.67	0.66 ± 0.72	1.94 ± 0.58

N is the sample size. A_t1_, A_t2_, A_t3_ have no units. Estimates are mean and jackknife standard error of the mean.

**TABLE 4:** Estimation of parameters, by age.

Age (yr)	Sex	*N*	*A*_t1_	*A*_b_	*A*_t2_	*T*_t1_	*T*_*b*_	$T_{\text{t}2}^{*}$	*W*_t1_	*W*_b_	*W*_t2_
20–29	F	994	1.9	−2.7	1.6	2	11.4	25.8	5	8.8	32.8
30–39	F	1,627	1.8	−2.1	1.7	2	11.6	27.4	5	9.4	33.2
40–49	F	1,305	1.4	−1.8	1.6	1.8	12	27	4.8	10	35.6
50–59	F	842	2.3	−0.8	2.4	2.4	14.6	37.8	6	7.4	48
≥60	F	364	2	−1.6	2	2.6	15.6	38.6	7.6	6.8	53
20–29	M	178	3.5	−1.6	2.5	2.6	11.8	33.8	6.4	7	44
30–39	M	392	3.7	−0.4	2.7	2.4	14.2	27.6	6.8	3.2	55.6
40–49	M	370	3.4	−0.2	2.4	2.6	16.6	28.8	9.4	2	43.8
50–59	M	275	3	0.6	3.7	3	17	28.8	13.2	-	36
≥60	M	175	3.8	1	3.5	5.6	15.6	27.6	12.4	-	53.8

* Values of T_t2_ are only approximate due to noise and the large width of the second relative tachycardia peak. T_t1_, T_*b*_, T_t2_, W_t1_, W_b_, W_t2_ are measured in days.

**TABLE 5:** Estimation of parameters, by severity.

Severity	Sex	*N*	*A*_t1_	*A*_b_	*A*_t2_	*T*_t1_	*T*_*b*_	$T_{\text{t}2}^{*}$	*W*_t1_	*W*_b_	*W*_t2_
Severe	F	221	4	−0.9	4.4	5	16.4	38.4	10	3.8	46.8
Severe	M	81	9.1	0.8	6.4	9.2	18.2	37.4	11.8	-	51.2
Mild	F	4,943	1.7	−1.8	1.5	2	12.4	28.2	5.2	9.6	37
Mild	M	1,326	3.3	0	2.7	2.8	15	28.2	7.8	-	41.4
Asymptomatic	F	452	0.7	−0.7	1.2	0	5	21.8	3.2	8.6	35.6
Asymptomatic	M	142	0.6	−0.7	0.8	1	8.8	27	5.2	5.4	21.2
Flu	F	360	2.6	−0.6	0.96	2.4	11	26.8	6.4	4.6	29.2
Flu	M	103	4.3	0.66	1.45	2.8	10.2	15.6	8.4	-	59.6

* Values of T_t2_ are only approximate due to noise and the large width of the second relative tachycardia peak. T_t1_, T_*b*_, T_t2_, W_t1_, W_b_, W_t2_ are measured in days.

### 3.2 Relative Bradycardia and Relative Tachycardia

Let us now quantify the prevalence of relative bradycardia and relative tachycardia. To do this, we define the following 4 windows each of which are 15 days long (starting and ending dates inclusive, and *D*_0_ is the date when symptoms present, only symptomatic individuals included):

• “Control” window from *D*_−45_ to *D*_−31_.
• Presumed “healthy” window from *D*_−30_ to *D*_−16_.
• Presumed “relative bradycardia” window from *D*_+7_ to *D*_+21_.
• Presumed “relative tachycardia” window from *D*_+22_ to *D*_+36_.

The healthy and control windows are expected to contain data when participants are healthy. The RHR is averaged over each window, and we only consider participants who have 15 days of data in each window. Let ΔRHR be the difference between the RHR averaged over window *w* and the RHR averaged over the control window:

$$\Delta RHR=\langle RH{R\rangle }_{w}-\langle RH{R\rangle }_{\text{control}}.$$ (4)

**FIGURE 5:**
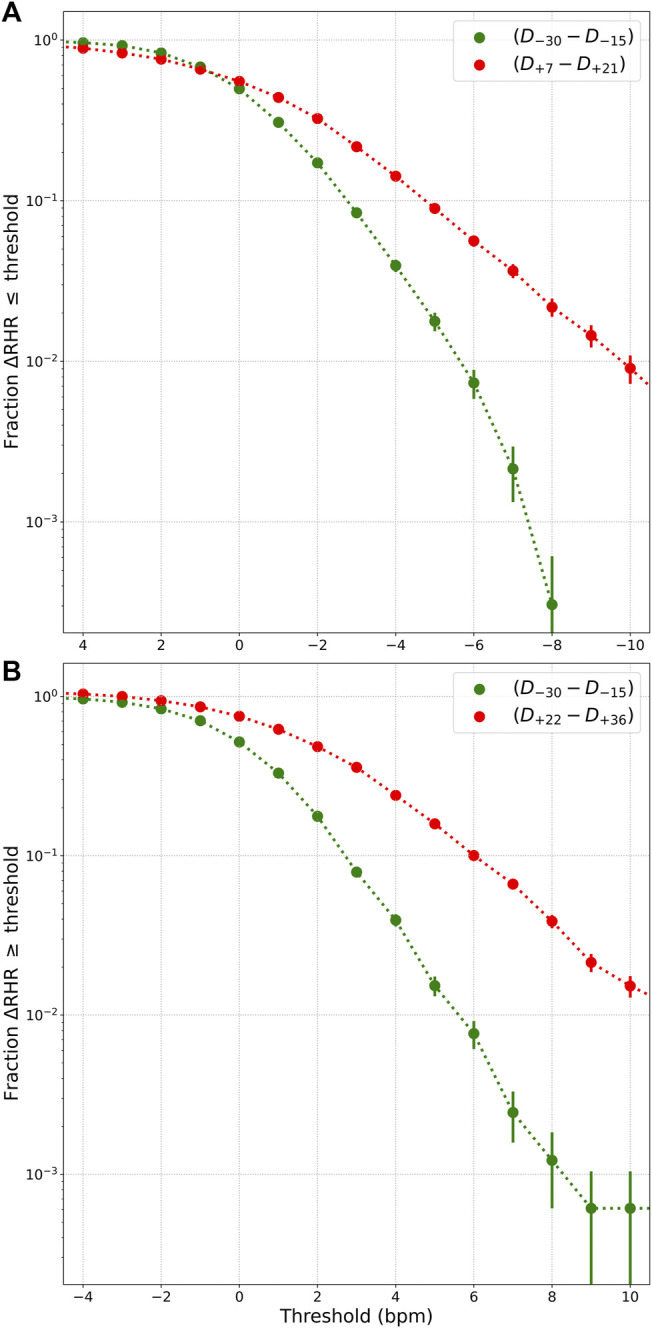
**(A)** Fraction of individuals with ΔRHR ≤x in the relative bradycardia window (red points), where *x* is a threshold, compared to the fraction that could be expected due to chance (green points). **(B)** shows the fraction of individuals with ΔRHR ≥x in the relative tachycardia window (red points), compared to the fraction that could be expected due to chance (green points). ΔRHR is the excess RHR compared to the control window. Error bars show standard error of the mean.

We compute the fraction of participants who have ΔRHR ≤x bpm, computed from the bradycardia window, and compare it to the same fraction computed from data in the healthy window, where *x* is a threshold value plotted along the horizontal axis. We do a similar comparison with the data in the tachycardia window. We compute the fraction of participants with ΔRHR ≥x computed from the tachycardia window, and compare with the fraction computed from data in the healthy window. Figure 5 shows this comparison. The curve plotted in red in Figure 5A shows the fraction of individuals in the time window *D*_+7_ − *D*_+21_ with ΔRHR ≤x plotted along the horizontal axis. This should be compared with the curve plotted in green which is an estimate of how likely it is that such a low ΔRHR may occur due to random chance. Figure 5B shows a similar comparison, but for ΔRHR ≥x , and considering the tachycardia window.

**FIGURE 6:**
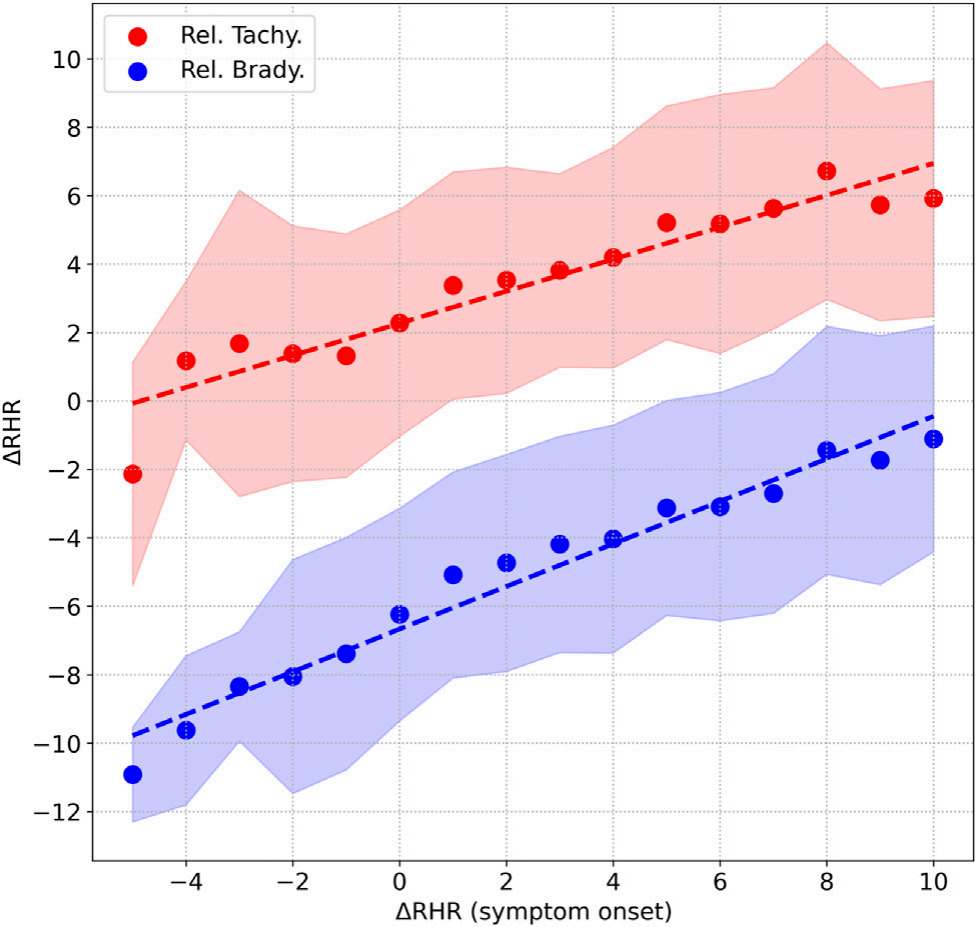
Correlation between the peak value of ΔRHR measured during symptom onset, and 1) peak value of ΔRHR in the second relative tachycardia window shown in red, 2) minimum value of ΔRHR measured in the relative bradycardia window shown in blue. The shaded areas represent the 1 standard deviation range.

Interestingly, there is a correlation between the peak value of ΔRHR measured during symptom onset (we use a 15 days window from *D*_−5_ to *D*_+9_) and the peak and minimum values of ΔRHR measured in the second relative tachycardia window (*D*_+22_ to *D*_+36_) and the relative bradycardia window (*D*_+7_ to *D*_+21_). Figure 6 shows this correlation. The horizontal axis is ΔRHR during symptom onset, while the vertical axis shows ΔRHR during either the second tachycardia phase (red) or the bradycardia phase (blue) (the points are the mean values, while the shaded contours show the one standard deviation range). We see that ΔRHR_tachy_ measured in the second relative tachycardia phase is positively correlated with ΔRHR_symptom_, the peak value observed at symptom onset. Similarly, ΔRHR_brady_, the minimum value observed during the relative bradycardia phase is positively correlated with ΔRHR_symptom_. This means that a low ΔRHR at symptom onset is indicative of a decreased RHR in the relative bradycardia phase. An approximate estimate of ΔRHR_tachy_ and ΔRHR_brady_ may be obtained by the following linear relations (with all measurements in bpm):

$$\begin{array}{ccc} \Delta {RHR}_{\text{tachy}} & = & 2.27+0.468\;\Delta {RHR}_{\text{symptom}}\\ \Delta {RHR}_{\text{brady}} & = & -6.67+0.622\;\Delta {RHR}_{\text{symptom}}, \end{array}$$ (5)

where we obtained the intercept and slope by fitting a linear line to the mean values.

### 3.3 Comparison With Other Measurements

**FIGURE 7:**
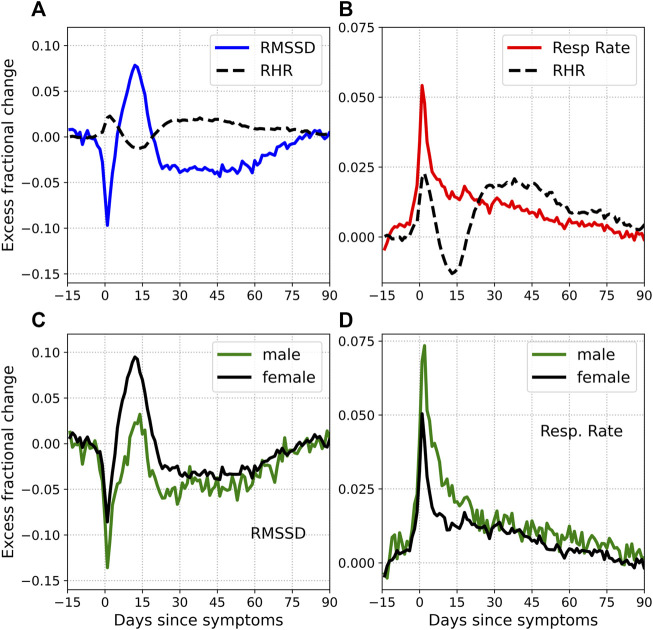
Excess fractional change in RMSSD and respiratory rate. Subplots **(A)** and **(B)** also show the excess fractional change in RHR (dashed black lines). Note that the RHR waveform is smoothed over time, resulting in a more rounded peak. Subplots **(C)** and **(D)** show the RMSSD and respiratory rate for male (green) and female (black) subjects respectively.

Let us now briefly consider 2 more health metrics: the heart rate variability quantified by the RMSSD and the respiratory rate. Figure 7 shows the excess fractional change Δ*ξ* in RMSSD [subplot (a)] and the respiratory rate [subplot (b)], plotted for symptomatic COVID-19. Also shown for comparison in dashed lines is the fractional change in RHR. The fractional change in RMSSD shows a similar time evolution as the RHR, except with opposite phase, i.e., when the RHR is elevated, the RMSSD is decreased and vice-versa. The relative bradycardia phase is seen to be accompanied by elevated heart rate variability, while the second relative tachycardia phase is associated with reduced heart rate variability. The variation is also of greater amplitude with the RMSSD compared to the RHR. The fractional change in the respiratory rate, on the other hand, does not show a similar time evolution. The respiratory rate is elevated during symptom onset, in response to the illness. It then decreases sharply, and then gradually returns to normal. Thus there is no equivalent of the relative bradycardia and second relative tachycardia phases with the respiratory rate.

## 4 Discussion

The growing popularity of wearable devices such as smart watches and trackers, and their increasing capability in measuring health biometrics makes them important tools in the field of digital health. From a national survey of 4,551 respondents conducted from January 2019 − April 2019, it was estimated that about 30% of adults in the United States use wearable devices, and over 82% are willing to share their health data with their care providers (Chandrasekaran et al., 2020). Telehealth can facilitate access to care, reduce the risk of transmission of COVID-19, and reduce strain on health care capacity and facilities (Demeke et al., 2021). Smartwatches and trackers that measure biomarkers that respond to illness such as heart rate, heart rate variability, respiratory rate, oxygen saturation, etc. can provide valuable information to health care providers. The market size for global wearable medical devices was nearly 30 billion in 2019, and is projected to reach ∼ 196 billion by 2027 (Fortune Business Insights, 2020). The COVID-19 pandemic has accelerated the popularity of telemedicine, with telehealth visits increasing from approximately 840,000 in 2019, to 52.7 million in 2020 (Suran, 2022).

A persistent problem with COVID-19 is the appearance of “long COVID” or “post COVID” symptoms for weeks or months after acquiring a COVID-19 infection, and can affect a whole spectrum of people, from those with very mild disease, to the most severe (Greenhalgh et al., 2020; Crook et al., 2021; Raveendran et al., 2021). It can also affect multiple organ systems, and can present with a wide array of symptoms (Greenhalgh et al., 2020; Crook et al., 2021; Raveendran et al., 2021). It is estimated that 10% of patients who test positive for COVID-19 remain unwell beyond 3 weeks (Greenhalgh et al., 2020). A complication to effective treatment for long COVID is that patients usually test negative for COVID-19 (Raveendran et al., 2021), yet show symptoms. Long COVID or post COVID thus refers to the time between microbiological recovery and clinical recovery (Raveendran et al., 2021). Long COVID might be considered post-acute COVID if symptoms persist beyond 3 weeks but less than 12 weeks, and chronic COVID when symptoms exist beyond 12 weeks (Greenhalgh et al., 2020). It is therefore not surprising that physiological biomarkers are affected for several months following a COVID-19 diagnosis. Wearable devices can play an important role in monitoring long COVID by measuring biomarkers indicative of illness. Radin et al. (2021) used data from Fitbit devices to show that the RHR is affected for 2–3 months following symptom onset. In this article, we confirmed the findings of Radin et al. (2021) using a large dataset of 7,200 COVID-19 positive and 463 flu positive individuals. Consistent with the findings of Radin et al. (2021), we find that the RHR can be modified for up to 3 months following symptom onset. Interestingly, this phenomenon is also seen in cases of flu. Both COVID-19 and flu cases show 3 distinct phases:

• First relative tachycardia phase during symptom onset when the RHR is elevated above normal, reaching a local peak value *A*_t1_ at a time *T*_t1_ days after symptom onset, with a full width at half maximum *W*_t1_. The peak value *A*_t1_ is higher in males compared to females, for COVID-19 (*p* − value < 0.0001) and flu (*p* − value = 0.0118).
• Following this peak, the RHR decreases, and reaches a local minimum value *A*_b_ at a time *T*_b_ days after symptom onset. If *A*_b_ < 0, we refer to this phase as relative bradycardia, and define a full width at half minimum *W*_b_. The condition *A*_b_ < 0 is satisfied for females (averaged over all ages), for COVID-19 (*p* < 0.0001), and for flu (*p* = 0.042). The condition *A*_b_ < 0 is not statistically significant for males (averaged over all ages), for either COVID-19 or flu.
• Following the minimum, the RHR increases, reaching a second local maximum *A*_t2_ at a time *T*_t2_ days from symptom onset, with a full width at half maximum *W*_t2_. The peak value *A*_t2_ is higher in males compared to females, for COVID-19 (*p* < 0.0001) and flu (*p* = 0.0667).

Figure 3 shows the fractional change in RHR *ξ* for flu and COVID-19, along with the control. Subtracting the variation for the control group allows us to calculate the effect due to illness Δ*ξ*. Figure 4 shows Δ*ξ* for male and female participants for different cases of COVID-19 severity, and for flu. Since these phases occur with both COVID-19 and flu, they are not novel to COVID-19. They can however, be more prominent in the case of COVID-19. It should be noted that flu has also been associated with cardiovascular disease (Nguyen et al., 2016). It is also known that the autonomic nervous system plays a key role in sensing and responding to infection, with pathogens activating vagal signaling causing bradyarrhythmias (Fairchild et al., 2011). Dysregulation of autonomic function has been reported in the weeks/months following a COVID-19 infection (Baker et al., 2022). It is intriguing that we see a statistically significant difference between male and female individuals, in the time variation of the RHR (Figure 4) and the time variation of the RMSSD (Figure 7). If the modification of the RHR and the RMSSD are indeed signs of autonomic dysregulation, it is intriguing that there is a statistically significant dependence on sex.

The parameters that describe the time evolution of the RHR depend on sex, age, and disease severity and are tabulated in Tables 3–5. The relative bradycardia minimum *A*_b_ and the time to the minimum *T*_b_ are correlated with age (The Pearson *r* for the correlation between *A*_b_ and age was found to be *r* = 0.80 for females and *r* = 0.97 for males. The Pearson *r* for the correlation between *T*_b_ and age was found to be *r* = 0.98 for females and *r* = 0.77 for males). The width of the first relative tachycardia peak *W*_t1_ is also correlated with age (*r* = 0.83 for females, and *r* = 0.94 for males).

Among the 3 phases, the relative tachycardia at symptom onset is the easiest to understand, and may be interpreted as an immune response to infection. More unexpected is the decrease in RHR, possibly resulting in transient relative bradycardia. Approximately 1% of individuals experience a value of RHR that is at least 10 bpm lower than normal (see Figure 5). Sinus bradycardia is known to occur during sleep in the case of COVID-19 (Hu et al., 2020). Since Fitbit’s measurement of RHR is not the lowest value of heart rate, this raises the possibility that the heart rate during sleep may reach dangerously low values. It is important to be aware of this transient relative bradycardia phase, since certain medications used in the treatment of COVID-19 such as Remdesivir have been known to cause bradycardia (Gupta et al., 2020; Jacinto et al., 2021; Sanchez-Codez et al., 2021).

Interestingly, mild cases of COVID-19 are more likely to present with relative bradycardia. It is also more likely to present in females compared to males. A possible explanation for this may be obtained from Figure 6 and Table 1. Figure 6 shows that the peak RHR during symptom onset is positively correlated with the magnitude of the dip, i.e., a smaller peak RHR during symptom onset is associated with a larger (and possibly negative) dip. It is well known the heart rate is strongly linked to temperature, increasing by 8.5 bpm per 1°C increase in temperature, as found in one study (Karjalainen and Viitasalo, 1986). As seen from Table 1, males are more likely to present with a fever compared to females. Similarly, fever is more common in severe cases compared to mild cases. Thus, females as well as mild cases presenting with a small RHR increase (or no increase) are more likely to experience a bradycardia dip compared to males, or severe cases. Similarly, Figure 6 shows that the peak RHR during symptom onset is positively correlated with the height of the second tachycardia peak. As a result, the second tachycardia peak is higher in males, and for severe cases.

It is interesting to ask whether heart rate variability is altered in a way similar to the RHR. The RMSSD reflects beat-to-beat variance in the heart rate and estimates vagally mediated changes (Shaffer and Ginsberg, 2017). We found that the RMSSD shows a similar time evolution as the RHR, except with opposite phase. Figure 7 shows the time evolution of the RMSSD [subplot (a)] and the respiratory rate [subplot (b)] with time. Unlike the RMSSD, the respiratory rate decreases monotonously past the initial peak. Subplots (c) and (d) show the variation of RMSSD and respiratory rate, for male and female subjects.

*Limitations:* There are several limitations to the current work. A major concern is that we do not know whether the individuals who were diagnosed with COVID-19 and flu received medications to treat their illness, and what effects those medications may have had on their heart rate. Nevertheless, treatment for COVID-19 (especially for mild presentations which are most commonly seen in our data) tends to be symptomatic, e.g., antipyretics to reduce fever, analgesic medications to relieve pain, etc. It is very unlikely that patients were prescribed medications weeks following a diagnosis. The symptoms, severity, as well as symptom start date were all obtained from a survey, and could not be independently verified. We have also assumed that the participants were healthy prior to being diagnosed with COVID-19 or flu, and that the control group consisted of healthy individuals. These concerns are partially mitigated by our large sample size so that a small number of outliers are not expected to skew results. An independent investigation by clinical personnel, of the resting heart rate and heart rate variability changes in the months following a COVID-19 diagnosis will yield further credibility to our findings. Despite these limitations, the work presented in this article shows how COVID-19 and flu can alter the resting heart rate in the weeks/months following infection. This knowledge can be crucial in the case of patients with cardiac complications, or when patients are being treated with medication known to have cardiac effects.

## Data Availability

The datasets presented in this article are not readily available because Fitbit’s privacy policy does not permit us to make the raw data available to third parties including researchers, outside of our web API Oauth 2.0 consent process. For specific questions, contact Fitbit at https://healthsolutions.fitbit.com/contact/.
